# Hematuria screening in patients undergoing lung cancer screening

**DOI:** 10.14440/bladder.2025.0008

**Published:** 2025-05-29

**Authors:** Jonathan Maldonado, Johnathan A. Drevik, Quinnlyn Walcott, Jacob Adams, Taylor Knowles, Helen Holzbeierlein, George Letner, Jeffrey M. Holzbeierlein, Elizabeth Wulff-Burchfield, Eugene K. Lee

**Affiliations:** 1 Department of Urology, School of Medicine, The University of Kansas, Kansas City, Kansas 66045, United States of America; 2 Department of Internal Medicine, School of Medicine, The University of Kansas, Kansas City, Kansas 66045, United States of America; 3 Department of Internal Medicine, University of Kansas, Kansas City, Kansas 66045, United States of America

**Keywords:** Hematuria screening, Urologic malignancies, Lung cancer screening, Smoking-related cancer risk

## Abstract

**Background:**

Bladder cancer, specifically urothelial carcinoma (UC), poses a significant health concern in the United States and is significantly influenced by tobacco use. Despite its prevalence, routine UC screening is not recommended due to diagnostic limitations and uncertain benefits in long-term survival.

**Objective:**

This study examined the effectiveness of urine dipstick screening for UC in subjects already at elevated risk for UC due to substantial smoking histories, who were undergoing low-dose computed tomography (LDCT) for lung cancer screening.

**Methods:**

A prospective study was conducted at a single academic center to screen patients undergoing annual LDCT for lung cancer screening for UC. Urine dipstick tests were performed on patients without a history of gross hematuria or previously diagnosed as having UC. Statistical analyses were used to evaluate the relationship between smoking history, urinalysis results, and the prevalence of urological malignancies.

**Results:**

We enrolled 201 patients with a mean age of 64.4 years and a balanced gender distribution. Urine dipstick tests detected red blood cells (RBCs) in 15% of patients, with 2.1% showing microhematuria on formal urinalysis (>2 RBC/high power field). Nine (4.5%) participants were advised to undergo comprehensive hematuria evaluations. In addition, four (2%) participants had a history or were newly diagnosed with urological malignancies (three bladder cancer and one kidney cancer).

**Conclusion:**

Urine dipstick testing during lung cancer screenings in patients with significant smoking histories may facilitate early detection of urological malignancies, potentially improving patient outcomes. Further research is required to validate these findings, determine cost-effectiveness, and develop standardized screening strategies.

## 1. Introduction

Bladder cancer, notably urothelial carcinoma (UC), stands as the sixth most prevalent cancer in the United States, with an estimated 80,000 new cases emerging annually, predominantly affecting males over 55 years of age.^1^ Tobacco use, the primary risk factor for UC, significantly elevates the risk of developing UC and accounts for about 50% of cases in men and 20% in women, a risk second only to that for lung cancer.^2^,^3^ Smoking heightens the risk of UC approximately three-fold compared to non-smokers, a risk that persists even after cessation and is subject to factors such as smoking duration and the time elapsed since quitting.^4^

Despite the clear criteria for effective cancer screening formulated by the National Cancer Institute, early disease detection, improved outcomes with early treatment, and a reduction in cause-specific mortality, routine screening for bladder cancer in the general population is not currently recommended.^5^ This is primarily due to the limitations of available diagnostic tools, such as urine cytology and biomarker assays, which have limited sensitivity and specificity, especially for early-stage and low-grade tumors.^6^,^7^ These diagnostic challenges lead to a high incidence of false positives and negatives, complicating clinical decision-making. In addition, the relatively low prevalence of UC in the general population questions the cost-effectiveness and practicality of widespread screening protocols.^8^ Moreover, there is a lack of conclusive evidence demonstrating that early detection through screening significantly improves long-term survival outcomes.^9^

In contrast, the United States Preventive Services Task Force (USPSTF) recommends lung cancer screening using low-dose computed tomography (LDCT) for individuals aged 50 – 80 years old who have a ≥20-pack-year smoking history, and currently smoke, or have quit within the past 15 years.^10^ Given this association between smoking and UC, there may be potential benefits in screening patients already identified to be at high-risk for lung cancer. This subgroup, characterized by their substantial smoking history, inherently carries an elevated risk for UC, suggesting that concurrent screening for both malignancies could be beneficial.^2^,^11^ Such an approach could lead to earlier detection and intervention of UC, improving patient outcomes in a population already under surveillance for lung cancer.

This study hypothesized that the significant smoking history in our cohort correlated with a higher prevalence of UC compared to the general population. We propose the adoption of urine dipstick screening as a cost-effective and non-invasive tool to facilitate early detection of UC in this group. The primary aim of the study was to assess the utility of incorporating UC screening through urinalysis (UA) in patients undergoing LDCT for lung cancer, potentially unveiling a higher incidence of occult urological malignancies in this high-risk population.

## 2. Methods

### 2.1. Study population

Institutional review board approval (IRB #142959) and patient informed consent were obtained for this prospective study. The study population comprised patients undergoing annual lung cancer screening through chest-LDCT at a single academic center. Eligible participants were those referred by their primary care providers for yearly cross-sectional chest imaging, specifically targeting individuals with a significant smoking history (defined as >20 pack-years of smoking history and age of 50 – 80 as described by the USPSTF). Inclusion criteria for this study encompassed individuals aged 18 years or older undergoing LDCT lung screening, with eligibility maintained even if they were participating in other research studies. We excluded patients with <20-pack-year smoking history or those with a history of anuria, defined as the absence of urine production.

### 2.2. Data collection

Upon presentation for LDCT, a comprehensive patient history was obtained. This included assessment of any previous episodes of gross hematuria, American Urological Association (AUA) symptom scores (AUASS), detailed smoking history, environmental exposures, family history, and any history of prior pelvic radiation, with interviews conducted uniformly across all patients. Patients reporting gross hematuria within the past year were advised to undergo a gross hematuria workup per standard clinical guidelines.

Urine dipstick tests were performed for all patients without a recent gross hematuria or previously diagnosed UC (The Multistix^®^ 10 SG reagent strips, Siemens Medical Solutions, USA). In cases where the urine dipstick was negative, no further urological workup was pursued. However, urine dipsticks indicating the presence of blood (moderate red blood cells [RBCs] or higher on urine dipstick) or infection (abnormal leukocytes or nitrites) were followed up with a micro UA or culture, respectively. We selected a threshold of “moderate RBCs on dipstick” (corresponding to a >50% color change on reagent strips) to optimize sensitivity while minimizing false negatives. This threshold was chosen based on prior studies suggesting it enhances detection rates for significant hematuria while maintaining reasonable specificity. In previous literature, the sensitivity of urine dipsticks for detecting microscopic hematuria ranged from 80 – 90%, with specificity varying between 65 – 85%, depending on the cutoff used. Future analyses incorporating sensitivity/specificity calculations specific to our cohort could further refine this threshold for clinical application.^12^ According to the AUA microhematuria guidelines, patients with more than 20 pack-year smoking history and microhematuria (defined as ≥3 RBCs per high-powered field [HPF]) were classified as high-risk subjects and were recommended to undergo further diagnostic procedures, including cystoscopy and cross-sectional urography.^13^

### 2.3. Statistical analysis

Statistical analyses were conducted to evaluate the characteristics and outcomes of patients undergoing screening for urological malignancies. Descriptive statistics were used to summarize the demographic and clinical characteristics of the study population, including mean ± standard deviation (SD) for continuous variables (age, body mass index [BMI], pack-year smoking history, and AUASS) and frequencies/percentages for categorical variables (gender, smoking status, history of pelvic radiation, and occupational exposure). Comparative analyses between patients with and without hematuria were performed using the chi-square test for categorical variables and independent *t*-tests for continuous variables. Statistical significance was set at *p*<0.05 for all two-sided tests. All statistical analyses were performed using the Statistical Package for Social Sciences for Windows, version 27.0 (IBM Corp., United States). The prevalence of pathological findings, such as urinary tract infections, gross hematuria, and the presence of RBCs on urine dipsticks, was calculated as a percentage of the total study population or the subset of patients who underwent specific tests. Finally, the prevalence of urological malignancies within the study population was determined and presented as a percentage.

### 2.4. Power

The study was designed with an appropriate power based on existing literature, which suggests that approximately half of the individuals with a significant smoking history present abnormal dipstick UA results.^4^,^13^ Previous research indicated a 1.2% incidence of urothelial cancer in patients with UA positive for microhematuria.^14^ Therefore, our study aimed to identify a prevalence of UC greater than 1% in the study cohort. Our study was designed with sufficient power to detect a prevalence of UC greater than 1% based on existing literature. While our sample size of 201 patients was modest, our findings demonstrated a notably higher prevalence of UC (2%). To further contextualize these results, a 95% confidence interval for the prevalence of UC in this cohort was calculated (0.3 – 4.7%), indicating that while our estimate is robust, larger studies are needed to refine prevalence estimates and assess the reproducibility of these findings. Statistical analyses were performed using appropriate statistical software, with significance levels set a priori, and data expressed as mean ± SD or percentages, as appropriate. The analysis included descriptive statistics for demographic data and inferential statistics to assess the association between smoking history, UA results, and the prevalence of urological malignancies.

## 3. Results

**Figure 1:**
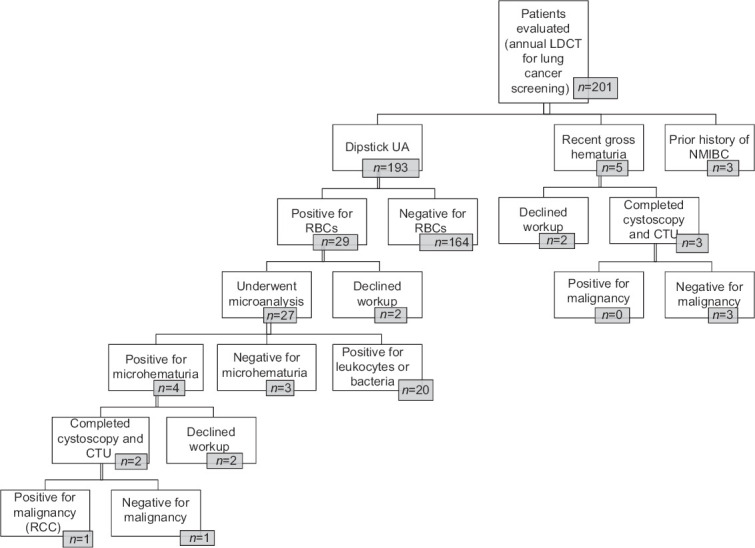
Flow chart of study cohort, urinalysis, completed workups, incidence and prevalence of malignancy, and outcomes Abbreviations: CTU: Computed tomography urography; LDCT: Low-dose computed tomography; NMIBC: Non-muscle invasive bladder cancer; RBCs: Red blood cells; RCC: Renal cell carcinoma; UA: Urinalysis.

This study evaluated 201 patients undergoing lung cancer screening for potential urological malignancies (Figure 1). The mean age of the cohort was 64.4 years (SD ± 6.51), with a balanced gender distribution of 100 females and 101 males. The average BMI was 30.26 kg/m² (SD ± 7.29). The cohort had a substantial smoking history, with a mean pack-year history of 45.91 (SD ± 21.01). Among them, 92 patients were present smokers, while 109 had quit smoking. The mean AUASS was 7.19 (SD ± 6.23). Three participants had a history of pelvic radiation. Occupational exposure to hazardous chemicals was reported by 30 patients, as detailed in Table 1.

**Table 1:** Patient demographics

Characteristic	Data
Age (mean [range], years)	64.4 (50 – 70)
Sex	
Female	100 (49.8)
Male	101 (50.2)
BMI (mean±SD, kg/m²)	30.26±7.29
AUASS (mean±SD)	7.2±6.23
Pack-year smoking history (mean±SD)	45.91±21.01
Active smokers	92 (45.8)
Former smokers	109 (54.2)
History of occupational exposure	30 (14.9)
History of pelvic radiation	3 (1.5)

Note: Data are presented as *n* (%) unless stated otherwise. Abbreviations: AUASS: American Urological Association symptom scores; SD: Standard deviation; BMI: Body mass index.

Of the 201 patients evaluated, five patients were found to have a history of gross hematuria, and three patients were found to have a history of non-muscle invasive bladder cancer. One patient was found to have renal cell carcinoma (RCC) as seen in Figure 1. Urine dipstick tests were conducted on 193 patients (96% of the cohort). Comparative analysis revealed no significant differences in age, gender, BMI, or smoking history between patients with and without hematuria (all *p*>0.05). Similarly, there was no significant difference in AUASS scores between hematuria-positive and hematuria-negative patients (*p*=0.32). However, patients with hematuria had a significantly higher rate of prior urological malignancy than those without hematuria (*p*=0.01). These findings suggest that while hematuria may be a marker for urothelial pathology, its utility as a primary screening tool warrants further investigation. Moderate RBCs on urine dipstick were detected in 15% (29/193) of the subjects, with 9.3% (18/193) showing isolated RBCs without other abnormalities. Further UA with microscopy in patients with moderate or higher RBC counts on dipstick confirmed microhematuria (≥ 3 RBCs/HPF) in 2.1% (4/193) of cases. Based on these findings and patients with recent gross hematuria, 4.5% (9/201) of the cohort were advised to undergo comprehensive hematuria evaluations (Table 2). Among the nine patients recommended for further hematuria evaluation, three were priorly diagnosed as having non-muscle invasive bladder cancer, and one patient was *de novo* diagnosed with RCC. Importantly, none of the four patients with confirmed microhematuria (≥3 RBCs/HPF) were newly diagnosed with UC. Gross hematuria was more strongly associated with known urological malignancies, as all three patients with prior UC had reported previous gross hematuria. In addition, 2.5% of patients had developed a pathological urinary tract infection necessitating oral antibiotics. Among the 29 patients with a positive urine dipstick for RBCs, 25 (86.2%) had no significant urological pathology. Conversely, one patient with RCC was negative for hematuria, indicating that while hematuria may be a useful screening tool, it does not capture all cases of urological malignancy. The age and risk factors, including smoking history, of the patients with urological malignancies are outlined in Table 3.

**Table 2:** Study cohort parameters and percentages

Parameter	Number of patients	Percentage of patients
Gross hematuria	5	2.5
NMIBC history	3	1.5
Patients undergoing a urine dipstick test	193	96.0
RBCs detected on urine dipstick	29	15.0
Isolated RBCs without other abnormalities	18	9.3
Confirmed microhematuria on microanalysis	4	2.1
Advised for comprehensive hematuria evaluation	9	4.5

Abbreviations: NMIBC: Non-muscle invasive bladder cancer; RBCs: Red blood cells.

**Table 3:** Patients with urological malignancies and associated risk factors

Patient	Malignancy	Gender	Environmental	Radiation	Pack-year smoking history (packs per day×years)
1	NMIBC	Male	-	-	96
2	NMIBC	Female	-	-	56
3	NMIBC	Female	-	-	66
4	RCC	Male	-	-	30

Abbreviations: NMIBC: Non-muscle invasive bladder cancer; RCC: Renal cell carcinoma.

## 4. Discussion

This study aimed to evaluate the prevalence of urological malignancies, particularly UC, in patients undergoing LDCT for lung cancer screening due to a significant smoking history. Despite promising early studies using chemical reagent strips for hemoglobin detection, the present lack of standardization in UC screening underscores a gap in urological cancer detection strategies.^15^

The global burden of UC, with varying incidence rates and etiologies, highlights the need for targeted screening approaches. Our study’s focus on a high-risk population, primarily smokers, is particularly relevant given the strong association between tobacco use and UC.^2^,^11^ We identified 5% (9/201) of patients in this cohort who warranted a comprehensive hematuria evaluation based on AUA guidelines. These patients would have otherwise been missed if not for UA dipstick screening. While our findings suggest that urine dipstick screening identified patients who would not have otherwise been referred for hematuria workup, it is essential to consider whether this occurred by chance or reflects a significant association. Our statistical analysis demonstrated that patients with hematuria had a significantly higher rate of prior urological malignancy (*p*=0.01), supporting the potential relevance of dipstick screening in high-risk populations. However, there were no significant differences in other demographic or clinical variables between hematuria-positive and hematuria-negative patients. Future studies with larger cohorts and multivariable analyses will be necessary to establish a causal relationship between hematuria detection and urological malignancies in this population. The history of a prior urological malignancy is relevant in this cohort because patients with a history of UC are at risk for recurrence, which can present as hematuria. While this study focused on incident UC diagnoses, our findings suggest that hematuria screening in patients with a prior history of UC may help detect recurrences earlier, reinforcing the importance of long-term surveillance. An important limitation of this study was the low compliance rate for follow-up hematuria evaluations, with only five out of nine (55.6%) patients completing the recommended workup. This reflects real-world challenges in adherence to additional diagnostic procedures, particularly in asymptomatic patients. Barriers such as patient anxiety, logistical constraints, financial concerns, and lack of symptom-driven urgency may contribute to low compliance rates. Addressing these issues would be critical for the feasibility of implementing a widespread urine-based screening program.

Previous studies by Messing *et al*.^15^ and Britton *et al*.^16^ have demonstrated the feasibility and potential effectiveness of bladder cancer screening using hematuria testing. These studies showed that screening can identify bladder cancers earlier, potentially improving associated treatment morbidity and survival rates. However, the positive predictive value of hematuria testing remains a concern, with many false positives leading to unnecessary evaluations and related costs.^17^ Our study also underscores the concern of false positives, which can lead to unnecessary invasive testing and increased healthcare costs. Improving specificity may involve refining cutoff thresholds for urine dipstick positivity, incorporating additional urinary biomarkers to enhance diagnostic accuracy, and utilizing risk stratification models that integrate patient history and exposure data. Combining urine dipstick screening with novel urinary molecular tests could also help distinguish benign hematuria from malignancy-associated hematuria, reducing unnecessary evaluations.

Our study contributes to this evolving landscape by highlighting the prevalence of urological malignancies in a high-risk population undergoing LDCT for lung cancer screening. The integration of urine dipstick testing in such screenings could potentially lead to earlier detection and improved management of urological malignancies, particularly in populations with significant smoking histories. There is increasing evidence of a shared pathophysiological mechanism between lung cancer and UC/RCC, primarily attributed to common carcinogenic exposures such as tobacco and occupational chemicals. While the co-occurrence of these malignancies has been observed in epidemiological studies, further research is needed to establish whether lung cancer screening populations harbor a significantly higher prevalence of UC/RCC. Similarly, head and neck squamous cell carcinoma shares common etiological risk factors with UC, particularly smoking and chemical exposure. However, the anatomical and histological differences in tumorigenesis between UC and head and neck squamous cell carcinoma suggest that direct screening implications for bladder cancer may be more specific to patients already undergoing radiological imaging for thoracic malignancies. For individuals with a significant smoking history, these findings underscore the importance of smoking abstinence or cessation, as well as the potential benefits of urine screening. These interventions should be offered at the same time as screening.

Our study also highlighted the incidence of pathological urinary tract infections and the presence of gross hematuria in this population. While urinary tract infections were relatively low (2.5%), oral antibiotics were prescribed for these patients; without urine screening, treatment would have been delayed. The identification rate of gross hematuria in this population was 2.5%, which underscores the importance of a comprehensive urological evaluation, especially in the context of a significant smoking history.

One of the limitations of our study is its single-center design, which may limit the generalizability of our findings. In addition, the reliance on patient-reported history for factors, such as smoking and environmental exposures could introduce recall bias. In addition, while we identified nine patients (4.5%) appropriate for hematuria workup, only five participants agreed. Our patient population reflects those undergoing LDCT for lung cancer screening at an academic medical center, which may not fully represent the broader community or rural settings. Geographic variations, healthcare access, and demographic differences in smoking habits could influence the applicability of these findings. Future multi-center studies are needed to assess whether similar trends in hematuria detection and urological malignancy prevalence are observed in diverse populations.

The incidence of urological cancers in this patient population was 2%, albeit most of these patients were diagnosed before participation in this study. We postulate that new diagnoses of UC would have been made for these individuals if not already identified before their inclusion in this study. Previous studies have reported that the incidence of microhematuria in the general population occurs at a rate between 10% and 13%, with approximately 1.2% of those affected ultimately diagnosed with UC.^14^,^18^ Based on these figures, screening roughly 1,000 patients would be required for one new diagnosis of UC. In contrast, this study found that only 70 individuals needed to be screened for one new diagnosis, supporting the utility of targeted screening in this high-risk population. While the concept of dual screening for lung and bladder cancer has potential benefits, it also presents ethical and logistical challenges. False positives can lead to unnecessary anxiety and invasive testing, which may not ultimately benefit patients. In addition, resource allocation, cost-effectiveness, and the psychological burden of incidental findings must be carefully considered. Future studies should assess the long-term impact of such screening programs on patient well-being, health system burden, and overall cancer mortality reduction. However, further studies are needed to determine if a clinical significance exists to warrant the economic demands of implementing such a screening program.

## 5. Conclusion

In conclusion, while UC screening is not yet standardized, our study supports the potential benefits of integrating urine-based screening methods in high-risk populations. This approach aligns with the criteria for effective screening and could lead to earlier detection, improved survival rates, decreased morbidity, and potentially more cost-effective management. Further research and large-scale prospective studies are needed to validate these findings and refine screening strategies for UC.

## Data Availability

Data will be made available upon reasonable request from the corresponding author.
